# A Novel Insecticidal Molecule Extracted from *Alpinia galanga* with Potential to Control the Pest Insect *Spodoptera frugiperda*

**DOI:** 10.3390/insects11100686

**Published:** 2020-10-11

**Authors:** Torranis Ruttanaphan, Georges de Sousa, Anchulee Pengsook, Wanchai Pluempanupat, Hannah-Isadora Huditz, Vasakorn Bullangpoti, Gaëlle Le Goff

**Affiliations:** 1 Animal Toxicology and Physiology Specialty Research Unit, Department of Zoology, Faculty of Science, Kasetsart University, Bangkok 10900, Thailand; torranis40@gmail.com (T.R.); fscivkb@ku.ac.th (V.B.); 2 Université Côte d’Azur, INRAE, CNRS, ISA, F-06903 Sophia Antipolis, France; georges.de-sousa@inrae.fr (G.d.S.); hannah.huditz@hotmail.de (H.-I.H.); 3 Department of Chemistry and Center of Excellence for Innovation in Chemistry, Faculty of Science, and Special Research Unit for Advanced Magnetic Resonance, Kasetsart University, Bangkok 10900, Thailand; a_ampere@hotmail.com (A.P.); fsciwcp@ku.ac.th (W.P.)

**Keywords:** 1′*S*-1′-Acetoxychavicol acetate, *Alpinia galanga*, *Spodoptera frugiperda*, Sf9 cells, botanical pesticide

## Abstract

**Simple Summary:**

The fall armyworm is an insect pest that feeds on many plants, including plants of agronomic importance, such as corn and rice. In addition, it has developed resistance to the main families of synthetic insecticides. There is, therefore, a need to find new, more environmentally friendly molecules to control this pest. We have extracted a molecule from greater galangal and tested its activity as an insecticide on the fall armyworm. This natural molecule causes larval growth inhibition and larval developmental abnormalities. To understand its action, a cell model with Sf9 cells was used. The molecule is much more toxic to insect cells than to human cells. It affects cell proliferation and induces cell death. This study demonstrates that a molecule extracted from an edible plant may have potential in the future development of botanical insecticides for the control of insect pests.

**Abstract:**

*Spodoptera frugiperda*, a highly polyphagous insect pest from America, has recently invaded and widely spread throughout Africa and Asia. Effective and environmentally safe tools are needed for successful pest management of this invasive species. Natural molecules extracted from plants offer this possibility. Our study aimed to determine the insecticidal efficacy of a new molecule extracted from *Alpinia galanga* rhizome, the 1′*S*-1′-acetoxychavicol acetate (ACA). The toxicity of ACA was assessed by topical application on early third-instar larvae of *S. frugiperda*. Results showed that ACA caused significant larval growth inhibition and larval developmental abnormalities. In order to further explore the effects of this molecule, experiments have been performed at the cellular level using Sf9 model cells. ACA exhibited higher toxicity on Sf9 cells as compared to azadirachtin and was 38-fold less toxic on HepG2 cells. Inhibition of cell proliferation was observed at sublethal concentrations of ACA and was associated with cellular morphological changes and nuclear condensation. In addition, ACA induced caspase-3 activity. RT-qPCR experiments reveal that ACA induces the expression of several caspase genes. This first study on the effects of ACA on *S. frugiperda* larvae and cells provides evidence that ACA may have potential as a botanical insecticide for the control of *S. frugiperda*.

## 1. Introduction

*Spodoptera frugiperda* (Smith) (Lepidoptera: Noctuidae), a major agricultural insect pest native to the American continent, has recently invaded Africa and Asia [1,2]. *S. frugiperda* causes severe yield losses in various species of economically important crops, such as maize, rice, cotton and peanut [3]. Synthetic insecticides are largely used for its management; however, their use has negative impacts on the environment and human health and led to the development of resistance. *S. frugiperda* has developed resistance against 41 different molecules (https://www.pesticideresistance.org/display.php?page=species&arId=200), resulting in pest control failures [4]. Such problems necessitate finding safer and innovative alternatives to the existing synthetic insecticides. Plants can be sources of organic molecules with insecticidal potential and advantages such as low toxicity to humans and environmental safety [5].

Plant secondary metabolites are organic compounds produced by plants and are known to play a role in adaptation to their environment [6]. They are usually subdivided according to their biosynthetic pathways in three main groups, including alkaloids, phenolics and terpenoids [6]. Plant secondary metabolites can act as insecticidal, antifeedant, repellency, oviposition deterrence and growth regulation agents against insects [5]. They have been intensively studied for the past 20 years to develop alternatives to synthetic insecticides due to their low ecological side effects [5,7].

*Alpinia galanga* (L.) Willd is a plant belonging to the Zingiberaceae family of Zingiberales order. Its rhizome has several active compounds, such as 1′*S*-1′-acetoxychavicol acetate (ACA), p-hydroxycinnamaldehyde, 1′-acetoxychavicol acetate, β-pinene, β-bisabolene and 1,8-cineole [8]. It has been used for various purposes, including antibacterial, antifungal, antiulcer, antitumor, antiallergic and antioxidant purposes. For example, it has been proposed as a new therapeutic agent in certain human cancers, such as myeloid leukemia [9], where it acts by inducing apoptosis, a natural occurring cell death process. ACA also has insecticidal activity against insect pests, such as *Bactrocera dorsalis* (Hendel), *Coptotermes gestroi* (Wasmann), *Coptotermes curvignathus* (Holmgren) and *Spodoptera litura* (Fabricius) [8,10,11,12]. Moreover, *A. galanga* oil could enhance the toxicity of a synthetic pyrethroid against *S. litura* [13]. In addition, *A. galanga* is a plant widespread in South East Asian countries and can be found throughout the year [14].

The aim of this study was to determine whether a compound extracted from *A. galanga*, 1′*S*-1′-acetoxychavicol acetate (ACA), could be used as a botanical insecticide against a major agricultural pest, *S. frugiperda*. It was a good candidate as it is a major constituent of *A. galanga* and its stereochemistry exhibited insecticidal activity [15]. The effects of this molecule and its possible mode of action were then investigated in the cell model of Sf9 cells by biochemical and molecular approaches.

## 2. Materials and Methods

### 2.1. Chemicals

Azadirachtin (95% purity), dimethyl sulfoxide (DMSO), phosphate-buffered saline (PBS), RNase A, propidium iodide (PI), penicillin-streptomycin, trypan blue solution (0.4%) and 3-(4,5-Dimethyl-2-thiazolyl)-2,5-diphenyl-2H-tetrazolium bromide (MTT) were purchased from Sigma-Aldrich, Steinheim, Germany. Alanyl-glutamine, trypsin/EDTA and fetal bovine serum were purchased from Sigma-Aldrich, St. Louis, MO, USA.

### 2.2. Extraction and Isolation of 1′S-1′-Acetoxychavicol Acetate

**Figure 1:**
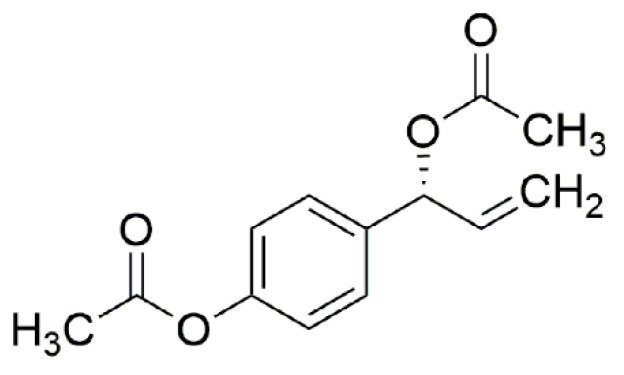
Chemical structure of 1′*S*-1′-acetoxychavicol acetate.

Sun-dried *A. galanga* (1.6 kg) rhizomes were powdered and sequentially extracted with hexane and ethyl acetate by soaking at room temperature for seven days. Ethyl acetate crude extract was filtered using a Buchner funnel and evaporated by a rotary evaporator. Ethyl acetate crude was fractionated by column chromatography (Kiesel gel 60 G cat No. 7731, Merck, Molsheim, France) with a gradient elution system of hexane-ethyl acetate (7:1). Eleven fractions (Fr. I–XI) were obtained. Fr. VI (250 mg) was purified using thin layer chromatography (Silica gel 60 PF254, Merck) with hexane and ethyl acetate. 1′*S*-1′-acetoxychavicol acetate (Figure 1) was obtained and verified by spectral analysis. The analytical data of 1′*S*-1′-acetoxychavicol acetate are described as below. High resolution mass spectra were recorded using a Maxis Bruker spectrometer (Karlsruhe, Germany). ^1^H and ^13^C NMR spectra were recorded using a Bruker 400 MHz Advance III HD spectrometer (Karlsruhe, Germany).

${\left[\alpha \right]}_{D}^{20}$ − 50 (*c* = 0.5, dichloromethane). ^1^H NMR (CDCl_3_, 400 MHz), δ: 7.37 (2H, d, *J* = 8.7 Hz), 7.07 (2H, d, *J* = 8.7 Hz), 6.26 (1H, d, *J* = 5.7 Hz), 5.98 (1H, ddd, *J* = 17.1, 10.5, 5.8 Hz), 5.30 (1H, dt, *J* = 17.2, 1.3 Hz), 5.25 (1H, dt, *J* = 10.5, 1.2 Hz), 2.30 (3H, s), 2.11 (3H, s). ^13^C NMR (CDCl_3_, 100 MHz), δ: 170.0, 169.4, 150.7, 136.4, 136.3, 128.6, 121.7, 117.3, 75.8, 21.1, 21.0. HRMS (ESI) Calcd. for C_13_H_14_NaO_4_ 257.0790 ([M + Na]^+^), Found 257.0825.

### 2.3. Insect Rearing and Insecticide Treatments

*Spodoptera frugiperda* populations were provided by Dr. Emmanuelle d’Alençon (DGIMI, Université de Montpellier, INRAE, Montpellier, France). The larvae were fed ad libitum on an artificial diet [16] and maintained under controlled conditions at 24 °C and 65% relative humidity with a 16 h light: 8 h dark photoperiod as described earlier [17].

The effects of ACA on early third-instar larvae of *S. frugiperda* were determined by topical application. Serial dilutions (0.005, 0.01, 0.02 µg/µL) of ACA were prepared in acetone. Doses were applied to the dorsal thoracic region of each larva using a micropipette. Each larva received 1 μL of solution per treatment, with acetone alone as the negative control. In each experiment, 10 larvae/treatment in three replicates were used (*n* = 30 per treatment). Treated larvae were placed in a sealed plastic container with an artificial diet under controlled conditions as described above. Larval weight, abnormal development and mortality were recorded after 24, 48 and 72 h. Larvae that did not move after being prodded were considered dead. Abnormal larvae were observed as described by [18]. Relative growth rate was calculated based on the following formula described by [19] as:

Relative growth rate (%) = (Mean larval weight of treated group/Mean larval weight of control group) × 100%

### 2.4. Cell Culture

Sf9 cells (from Invitrogen), derived from the ovarian tissue of *S. frugiperda* pupa, were cultured in 75-cm^2^ cell culture flasks (TPP, Trasadigen, Switzerland) with the Insect-XPRESS^TM^ protein-free medium (Lonza, Verviers, Belgium) and routinely maintained at 26 °C in a cell culture incubator (Memmert GmbH & Co., Schwabach, Germany). Cells were subcultured after achieving 90% confluence, and cell media were changed every 4 days.

HepG2 cells, the human hepatocellular carcinoma cell line, were provided by Dr. Georges de Sousa (INRAE, Sophia Antipolis, France). Cells were routinely maintained in William’s E Medium (Sigma-Aldrich, Steinheim, Germany) supplemented with 1% alanyl-glutamine, 1% penicillin-streptomycin and 10% fetal bovine serum at 37 °C in a humidified atmosphere of 5% CO_2_. After achieving 80–90% confluence, the cells were washed using PBS (Sigma-Aldrich, Steinheim, Germany) and trypsinized with 0.25% Trypsin/EDTA. Cells were plated in 75-cm^2^ cell culture flasks (TPP, Trasadigen, Switzerland), and cell media were changed every 3 days.

### 2.5. Cell Viability Assay

For cell viability assays, Sf9 cells (5 × 10^5^ cells/mL) and HepG2 cells (2 × 10^5^ cells/mL) were seeded onto 96-well plates (100 µL/well). Sf9 cells were treated with 0, 0.25, 0.875, 1.125, 1.75, 2.625, 3.25, 4, 5, 7.5, 10, 12.5, 15, and 17.5 µM of ACA in DMSO while HepG2 cells were treated with 5, 10, 20, 40, 80, 160, and 320 µM of ACA. After 24 h of incubation, each well received 100 μL of solution mixed with medium per treatment, with 0.25% DMSO as the negative control and azadirachtin (0–100 µM), the pure molecule, as the positive control. After 24 h of treatment, 100 µL MTT dissolved in Insect-XPRESS^TM^ medium (0.5 mg/mL) was added to each well. After an additional 2 h of incubation, the medium was discarded and DMSO was added to dissolve the formazan crystal, and the absorbance was measured at 570 and 690 nm using a microplate reader (SpectraMax, Molecular Devices). (Wokingham, UK). The relative percentage of cell viability was calculated as:

Cell viability rate (%) = [(*A* − 0.02)/(*B* − 0.02)] × 100%

where *A* and *B* are the absorbances of the ACA treated cells and DMSO treated cells, respectively.

### 2.6. Cell Proliferation Assay

Sf9 cells (3.5 × 10^5^ cells/mL) were seeded onto 6-well plates (2 mL/well). Serial dilutions of 0.38, 0.57, 0.88 and 1.17 µM (Inhibitory concentration (IC) IC_5_, IC_10_, IC_20_ and IC_30,_ respectively) ACA were prepared using DMSO. After 24 h of incubation, each well received 2 mL of solution mixed with medium per treatment, with 0.25% DMSO as the negative control. After 24, 48 and 72 h of treatment, cell density was evaluated using a Malassez hemocytometer (Marienfeld, Harsewinkel, Germany) counted with 0.4% Trypan blue solution staining. The morphologic characteristics of Sf9 cells were observed with an inverted phase contrast microscope (Nikon Eclipse TE2000-U, Tokyo, Japan) at 72 h after 1.17-µM ACA treatment. To examine the reversible arrest of cellular proliferation, the medium containing ACA was removed after 72 h of treatment. The cells were washed using PBS and fresh medium without ACA was added. After an additional 72 h of incubation, the cells were counted.

### 2.7. Caspase-3 Activity Assay

Caspase-3 activity was measured using a fluorometric activity assay kit (Molecular probe, Eugene, OR, USA) following the manufacturer’s protocol. The substrate, rhodamine 110 bis-(*N*-CBZ-l-aspartyl-l-glutamyl-l-valyl-l-aspartic acid amide) (Z-DEVD-Rho 110), was cleaved proteolytically by caspase-3. After 24 h treatment with ACA (0.88, 1.17, and 1.75 µM corresponding to IC_20_, IC_30_ and IC_40_, respectively), the cells were harvested and lysed in 1 mL lysis buffer. The sample was incubated with the substrate Z-DEVD-Rho 110. Fluorescence intensity was determined using a LightCycler 480^®^ (Roche Diagnostic, Inc., Mannheim, Germany) at an excitation wavelength of 490 nm and an emission wavelength of 520 nm. Values were expressed as arbitrary units of Rhodamine-110 (Rho 110) fluorescence.

### 2.8. Hoechst 33342 Staining

Sf9 cells (1 × 10^5^ cells/mL) were seeded in 96-well plates, incubated for 24 h and then treated with ACA (0.88, 1.17, and 1.75 µM corresponding to IC_20_, IC_30_ and IC_40_, respectively) and incubated for 24 h. The cells were stained with Hoechst 33342 (Thermo Fisher Scientific Inc. Waltham, MA, USA) and incubated for 1 h at 26 °C in a cell culture incubator. The stained cells were observed using an ArrayScan™ XTI High Content Analysis (Thermo Fisher Scientific Inc. Waltham, MA, USA).

### 2.9. Cell Cycle Analysis

The Sf9 cell cycle analysis was carried out using fluorescence-activated cell sorting (FACS) assay (FACSCalibur, BectonDickinson, Eysins, Switzerland). The Sf9 cells were incubated for 24, 48 and 72 h with 1.17 µM of ACA (IC_30_). Cells were resuspended and fixed with cold 80% ethanol/PBS (10 mM Na_2_HPO_4_, 138 mM NaCl, 2.7 mM KCl, pH 7.4). Fixed Sf9 cells were incubated in a PBS solution containing 50 µg/mL propidium iodide and 50 µg/mL RNAse A at 37 °C for 20 min. At least 10,000 cells were counted in each assay. The percentage of cells in each cell cycle phase were measured and classified in G0/G1, S and G2/M phases, depending on the intensity of the fluorescence peaks. Cells incubated with 0.25% DMSO at the same time were used as a control.

### 2.10. RNA Extraction, cDNA Synthesis and Real-Time Quantitative PCR (RT-qPCR)

To study gene expression of apoptosis-related genes induced by ACA, Sf9 cells were harvested after 24 h exposure to 1.17 µM ACA corresponding to IC_30_. Total RNA was extracted using the TRIzol Reagent (Invitrogen Life Technologies, Carlsbad, CA, USA) according to the manufacturer’s protocol. RNA extractions were performed in six-well plates with three biological replicates.

An amount of 1 μg total RNA was reverse transcribed using the iScript cDNA Synthesis kit (Bio-Rad). RT-qPCR reactions were performed using an Opticon monitor 2 (Bio-Rad) with the qPCR MasterMix for SYBR^®^ Green I No ROX (Eurogentec, Seraing, Belgium). The PCR conditions were as follows: 50 °C for 2 min, 95 °C for 10 min, followed by 40 cycles of 95 °C for 30 s, 60 °C for 30 s and 72 °C for 30 s. Each reaction was performed in two technical replicates and the mean of the three independent biological replicates was calculated. All results were normalized to the glucose 6-phosphate dehydrogenase (G6PDH), ribosomal protein L4 (RpL4) and ribosomal protein L18 (L18) mRNA expression levels, identified, in one of our previous studies, as the most stably expressed reference genes under different conditions [20]. Relative expression values were calculated in R using the SATQPCR (http://satqpcr.sophia.inra.fr) described in [21]. Primer sequences and efficiencies are listed in Table 1.

### 2.11. Statistical Analysis

All experiments were conducted in three biological replications and the values are described as mean ± SE (standard error). Probit analysis was used to estimate contact toxicity as the median lethal dose (LD_50_) values. Statistical comparisons between various groups were analyzed using Tukey’s multiple range tests (*p* < 0.05 being considered significant) by the program Stat Plus v.Pro 6.5.0.0 (AnalystSoft Inc, Walnut, CA, USA).The half maximal inhibitory concentration (IC_50_) values were calculated by log-probit analysis using the GraphPad Prism 5 program. For RT-qPCR analysis, statistical comparisons between DMSO and ACA groups were analyzed using *t*-test by the SATQPCR (http://satqpcr.sophia.inra.fr/cgi/home.cgi), described in Rancurel et al. [21].

## 3. Results

### 3.1. Extract Yield

The percentage yield of *A. galanga* for each crude extract derived from 1.6 kg of dry material was measured, and we found that ethyl acetate crude extract resulted in the highest yield percentage (1.19% yield), followed by hexane crude extract (0.43% yield). ACA (156 mg) was derived from 1 g of ethyl acetate crude extract. Ethyl acetate crude extract was brown gum, while ACA was pale yellow solid.

### 3.2. Effects of ACA on S. frugiperda Larvae

**Figure 2:**
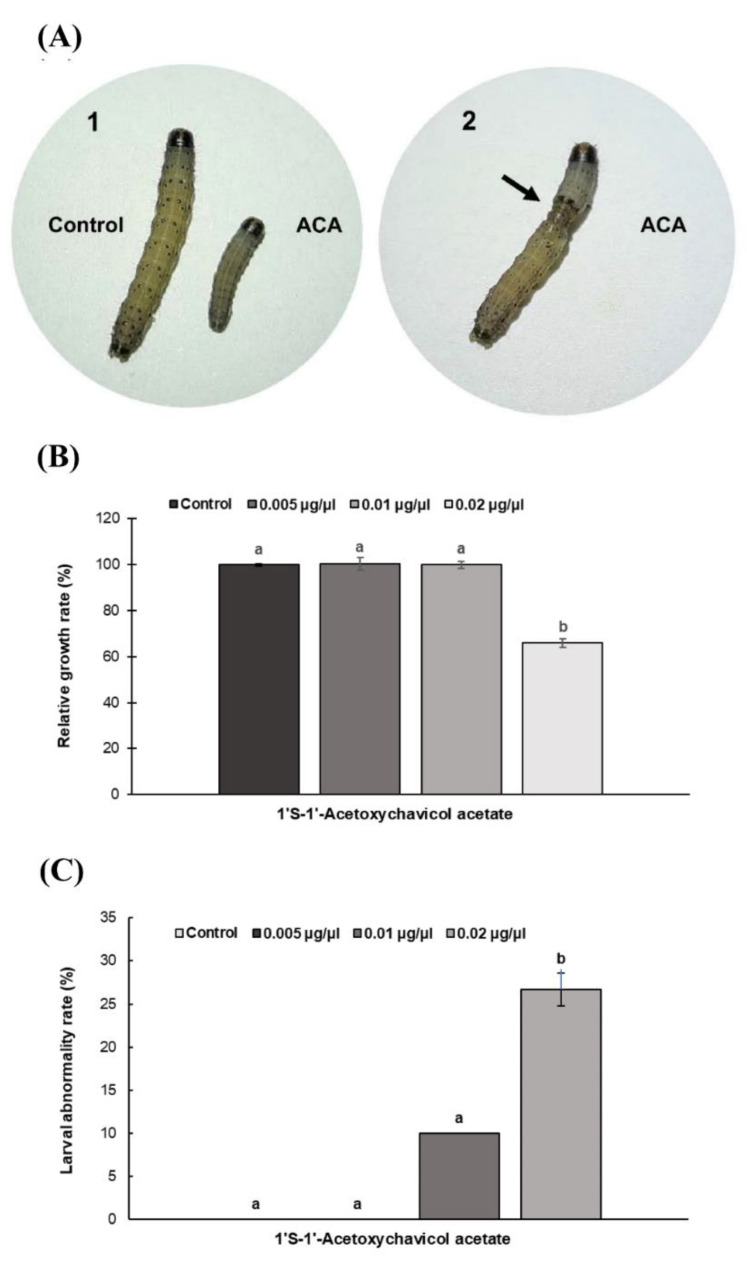
Effects of 1′*S*-1′-acetoxychavicol acetate (ACA) on *Spodoptera frugiperda* larvae; (**A**) Phenotypic changes of *S. frugiperda* larvae. (**A1**) Larval body size of *S. frugiperda* at 72 h after ACA treatment compared with acetone-treated larvae (negative control). (**A2**) Abnormalities of *S. frugiperda*. (**B**) Relative growth rate (%). (**C**) Larval abnormality rate (%). Arrow indicates larval abnormality region. Different lowercase letters indicate significantly different groups using Tukey’s honestly significant difference (HSD) test (*p* < 0.05). In each experiment, 10 larvae/treatment in three replicates were used (*n* = 30 per treatment).

The effects of ACA on the growth inhibition and abnormality phenotype of third instar larvae of *S. frugiperda* were determined. Larval weights and phenotypic changes were examined after treatment with ACA and acetone (negative control). After 72 h, the larvae treated with ACA exhibited growth inhibition and abnormal phenotypes, such as a shrunk body, wrinkled body, deformed body and parts of the old cuticle remaining in the ACA-treated region (Figure 2A). The size of ACA-treated larvae was obviously smaller as compared to the larvae of the negative control. Larvae treated with 0.02 µg/µL ACA showed a significant reduction in relative growth rate of 34% (*p* < 0.05, Figure 2B) and a significant increase in abnormal phenotypes of around 27% (*p* < 0.05, Figure 2C). Additionally, the larvae treated with 0.02 µg/µL ACA weighed significantly less, with a 31.79% decrease (13.55 mg ± 1.15 vs. 19.87 mg ± 1.19, *p* < 0.05, *n* = 30).

### 3.3. Cytotoxicity of ACA on Sf9 and HepG2 Cells

**Figure 3:**
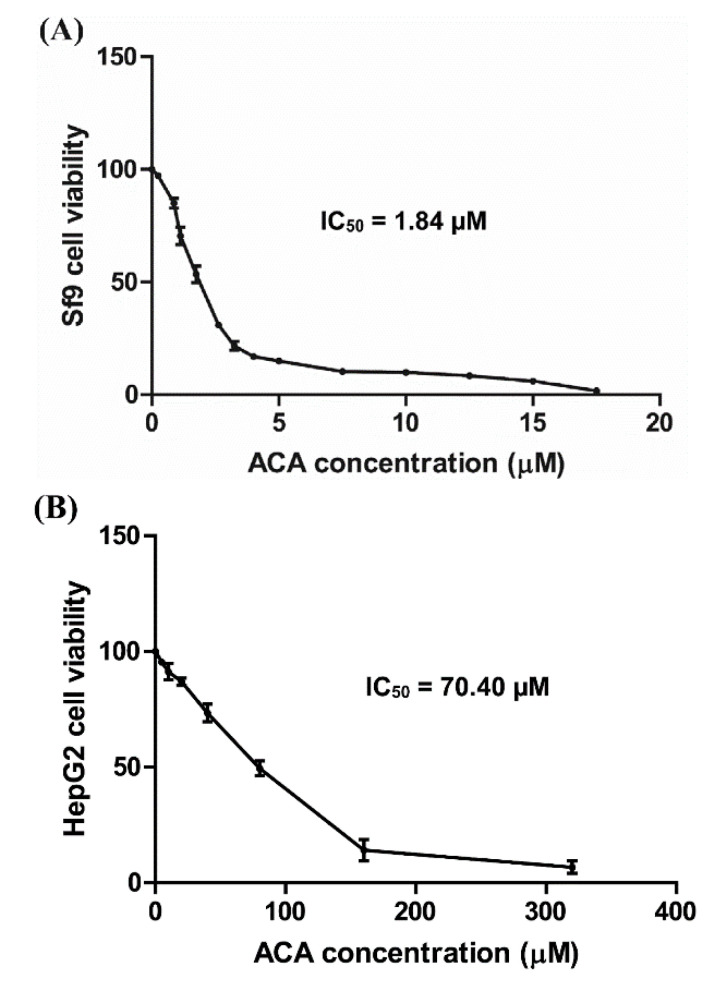
Effects of 1′*S*-1′-acetoxychavicol acetate on (**A**) Sf9 and (**B**) HepG2 cell viabilities at 24 h after treatment. IC_50_ represents the half maximal inhibitory concentration.

In order to further investigate the effect of ACA at the cellular level, we used a cellular model, Sf9, an *S. frugiperda* cell line. Cell viability was measured by MTT assay after 24 h of compound exposure. ACA exhibited a high toxicity on Sf9 cells in a dose-dependent manner (IC_50_ = 1.84 µM, Figure 3A), while azadirachtin (a commercial botanical pesticide used to control lepidopteran pests in particular), a positive control, was less toxic than ACA (percent inhibition of 100 µM azadirachtin = 11%). In order to explore the selective toxicity of ACA, experiments were performed on the non-target cellular model HepG2 cells. ACA was toxic on HepG2 cells in a dose-dependent manner (IC_50_ = 70.40 µM, Figure 3B), however this toxicity was 38-fold lower than the one observed on Sf9 cells.

### 3.4. Sf9 Cell Proliferation Is Inhibited by ACA

**Figure 4:**
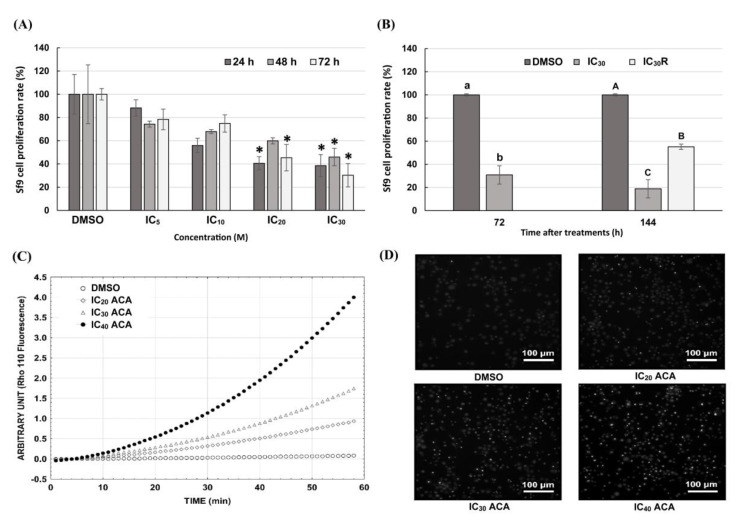
Effects of 1′*S*-1′-acetoxychavicol acetate on Sf9 cell proliferation, caspase-3 activity and nuclear condensation; (**A**) anti-proliferative effect of ACA at various concentrations and time points. (**B**) Sf9 cell proliferation was observed at 72 and 144 h after treatment by 1.17 µM of ACA. Reversible arrest of cell proliferation was observed in IC_30_R where the molecule was removed after 72 h. R stands for removal. All data were expressed as mean ± SE of three independent experiments. A *t*-test was performed to determine significance of the results as compared to the control group (0.25% dimethyl sulfoxyde DMSO). * denotes significant difference at *p* < 0.05. Tukey’s honestly significant difference (HSD) test was used to compare the groups of each time intervals. Different lowercase (for 72 h) or uppercase (for 144 h) letters indicate significantly different groups (*p* < 0.01). (**C**) Caspase-3 activity of Sf9 cells after 24 h treatment with ACA. Values were expressed as arbitrary units of Rhodamine-110 (Rho 110) fluorescence. (**D**) Nuclear morphologic changes of Sf9 cells was detected by Hoechst 33342 staining.

The effect of ACA on cell proliferation was examined by staining to assess the cell membrane integrity after treatment with sublethal concentrations (IC_5_, IC_10_, IC_20_ and IC_30_) of ACA, at various time intervals from 24 h to 72 h. The cell density in the DMSO control corresponds to the 100% proliferation rate set for each time. The proliferation rate of ACA-treated cells was calculated relative to this control. The cell density increased in the control as follows: 4.58 × 10^5^ cells/mL, 9.75 × 10^5^ cells/mL and 23.33 × 10^5^ cells/mL at 24, 48, and 72 h, respectively. All treatments showed a dose-dependent decrease in the rate of cell proliferation (Figure 4A). A significant inhibition of cell proliferation was observed in the concentration range between 0.88 and 1.17 µM, with no significant difference between proliferation rate at 24 h or 72 h. Cell densities were 1.62 × 10^5^ cells/mL, 4.83 × 10^5^ cells/mL and 7.10 × 10^5^ cells/mL at the concentrations of 1.17 µM after 24, 48 and 72 h of treatment, respectively. Inverted phase contrast microscopy observation revealed that Sf9 cells were irregular in shape, became larger in size and lost adhesion after 72 h treatment with 1.17 μM of ACA. In order to further confirm that ACA inhibited cell proliferation, the reversible arrest of cell proliferation was examined. The proliferation rate of the cells at 72 h after removal of ACA (1.17 µM) was significantly higher than the cells with the medium containing ACA, suggesting that the inhibitory effect of ACA on Sf9 cell proliferation is reversible (Figure 4B).

In order to evaluate the possible mechanisms involved the inhibition of cell proliferation, cell cycle monitoring and measurements of apoptosis-associated caspase activities were determined. The cell cycle was examined after 24, 48, and 72 h of 1.17 µM ACA treatments using FACS analysis. The distribution of cells in the different phases of the cell cycle was the same between control and ACA-treated cells (no significant difference) and no cell cycle arrest was observed, indicating that ACA did not affect the cell cycle (data not shown). Caspase-3 activity was evaluated in Sf9 cells after 24 h of IC_20_, IC_30_ and IC_40_ of ACA treatments (0.88, 1.17 and 1.75 µM, respectively) using Z-DEVD-Rho 110 (Figure 4C). The results demonstrated an increase in Rho 110 fluorescence following ACA treatments, indicating that ACA induced caspase-3 activity in Sf9 cells in a dose-dependent manner. Furthermore, Hoechst 33342 staining revealed nuclear morphologic changes (Figure 4D). Nuclei of ACA-treated cells showed deeper white staining with nuclear condensation after 24 h treated with 0.88, 1.17 and 1.75 µM of ACA. The number of nuclear condensations increased in a dose-dependent manner. Altogether, these results suggest that the inhibition of cell proliferation by ACA was not due to cell cycle arrest but appeared to involve apoptosis, as evidenced by caspase activity and nuclear condensation.

### 3.5. Gene Expression Profile of Apoptosis-Related Genes after ACA Treatment of Sf9 Cells

**Figure 5:**
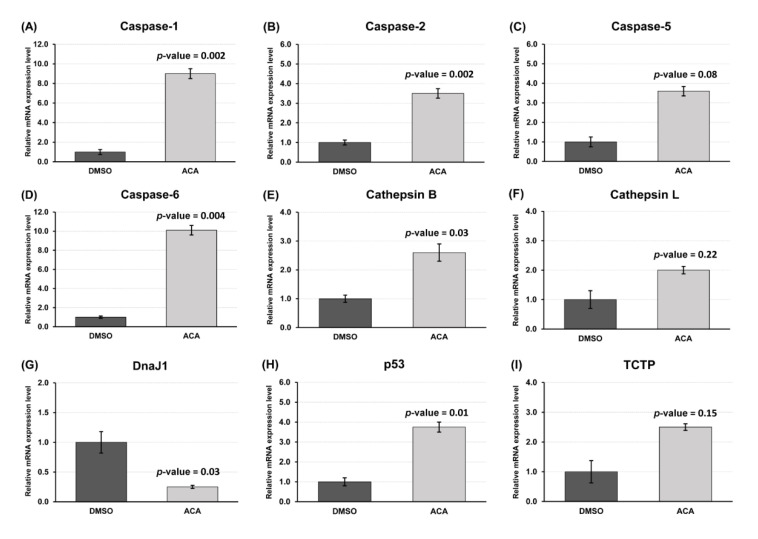
Effects of 1′*S*-1′-acetoxychavicol acetate on gene expression of Sf9 cells at 24 h after treatment; (**A**) Caspase-1 gene; (**B**) caspase-2; (**C**) caspase-5; (**D**) caspase-6; (**E**) cathepsin B; (**F**) cathepsin L; (**G**) DnaJ1 gene; (**H**) p53; (**I**) TCTP gene. Gene expression levels were normalized to the three reference genes; glucose 6-phosphate dehydrogenase (G6PD), ribosomal protein (L18) and ribosomal protein (RpL4). All data were expressed as mean ± SE of three independent experiments. A *t*-test was performed to determine significance of the results compared with the control group (DMSO-treated cells).

To investigate the effect of ACA at the transcriptional level, mRNA expression levels of nine genes that play important roles in apoptosis were measured by RT-qPCR. Of these nine genes, six showed a significant differential expression after ACA treatment (Figure 5). The expression of *caspases 1*, *2*, *6*, *cathepsin B* and *p53* was significantly up-regulated compared to the DMSO-treated group following exposure to 1.17 µM ACA for 24 h (Figure 5A,B,D,E,H), while DnaJ1 was significantly downregulated (Figure 5G). These results indicate that ACA was involved in apoptosis of Sf9 cells at the transcriptional level.

## 4. Discussion

Plant secondary metabolites with insecticidal properties have long been of interest as alternatives to synthetic insecticides for insect pest management [7]. Many plant secondary metabolites appear highly effective with multiple mechanisms of action while having low toxicity to non-target organisms [22,23]. So far, *A. galanga,* a plant belonging to the Zingiberaceae family, has been known for its wide range of medicinal properties [8] but little is known regarding its potential use as source of insecticide molecules. In our study, a major constituent, the 1′*S*-1′-acetoxychavicol acetate (ACA), was extracted from *A. galanga* and its insecticidal potency was evaluated on an insect pest, *S. frugiperda*.

The sustainable development of agriculture requires insecticides that are effective in insect pest management and less toxic to non-target organisms and humans [24]. ACA toxicity was evaluated in parallel on insect cells (Sf9) and mammalian cells (HepG2). HepG2 are duman hepatocellular carcinoma cells that have retained many activities found in normal hepatocytes [25,26]. Hepatocytes are increasingly being used as a cellular model for assessing the toxic potential of substances, as the liver is the principal organ associated with metabolic processes and xenobiotic toxicity [27]. Many insecticides have been tested on HepG2 cells to assess their effects on non-target organisms [28,29]. In our study, ACA was significantly less toxic to HepG2 cells than to Sf9 cells, consistent with what is expected in terms of selectivity for an insecticide molecule.

The ACA effects on the third-instar larvae of *S. frugiperda* showed an inhibition of growth and phenotypic abnormalities, such as a shrunk body, wrinkled body, deformed body and the remaining of parts of the old cuticle in the ACA-treated region. These effects are similar to those observed with insect growth disruptors, such as juvenile hormone analogues, inhibitors of chitin synthesis and ecdysone agonists. These chemicals interfere with the molecular, biochemical and physiological processes associated with insect growth and development [30,31,32]. However, similar mechanisms of inhibition of insect growth have also been observed in insects exposed to plant secondary metabolites. Indeed, azadirachtin is a tetranortriterpenoid limonoid isolated from *A. indica* and it could regulate growth and cocooning in *Bombyx mori* L. by inducing apoptosis in the prothoracic gland [33]. Furthermore, Shu et al. [34] reported that azadirachtin inhibited the larval growth of *S. litura* by inducing apoptosis in the midgut cells (including intestinal wall cracking, abnormal cell structure and cell death). In order to investigate the mechanisms involved in this growth inhibition, ACA effects have been evaluated on the cellular model of *S. frugiperda*, the Sf9 cells. Our results indicated that ACA had a higher toxicity than the widely used botanical insecticide azadirachtin. At sublethal concentrations, ACA caused inhibition of cell proliferation. The observed effects, including changes in cell morphology, nuclear condensation and induction of caspase-3 activity, suggested that the inhibition of cell proliferation is due to apoptosis. Similar effects of another plant compound, harmine, isolated from *Peganum harmala* were observed on Sf9 cells [35]. The apoptosis in this case had been highlighted by DNA fragmentation, cellular morphological changes, nuclear condensation, and increased caspase-3 activity [35]. Apoptosis is a naturally occurring cell death process responsible for the elimination of damaged or unwanted cells to maintain homeostasis and normal development in multicellular organisms in response to stimuli such as heat, hormones, hypoxia, radiation, virus and chemical agents [36]. Apoptosis is regulated by multiple proteins, such as caspases, inhibitors of apoptosis proteins (IAP) (including IAP, IAP-2, Survivin and Survivin-1), IAP antagonist proteins (including IBM1 and Grim-19), anti-apoptotic proteins (including DnaJ1 and TCTP), pro-apoptotic proteins (including Cathepsin B and Cathepsin L), p53 protein, etc. [37]. Among these proteins, caspases (Cysteinyl aspartate specific proteinase) form a family of cysteine proteases playing important roles in programmed cell death and can be classified, according to their biological functions, into two groups—initiator and effector caspases [38]. Once activated, initiator caspases initiate the apoptosis processes and effector caspases cleave various cellular substrates, ultimately leading to apoptosis [39]. Six caspases of lepidopteran insects have been classified based on phylogenetic analyses (caspase-1 to -6) [40]. Among them, caspase-3 is known as an effector caspase in apoptosis because of its pivotal roles, such as catalyzing the cleavage of various cellular proteins during apoptosis, coordinating the dismantling of cellular structures, chromatin condensation, DNA fragmentation, cellular morphological changes and the formation of apoptotic bodies [36]. The differential expression of caspases after exposure to chemicals or pathogen infection illustrated their various responses to external stimuli [41]. Moreover, the apoptosis, process also occurs via caspase-independent apoptotic pathways. For example, azadirachtin can induce mitochondria-mediated apoptosis by causing the opening of mitochondrial permeability transition pores and loss of mitochondrial membrane potential, ultimately leading to the release of cytochrome-c in the cytoplasm and activating caspases in the Sf9 cells [42]. The lysosomal signaling pathway also plays a role in the process of apoptosis. It was shown that azadirachtin induced lysosomal membrane permeabilization, cathepsin L releasing to cytosol and activation of caspase-3 in Sf9 cells [43]. The induced apoptosis in insect cells will help to elucidate the inhibition mechanism of cellular proliferation that could also inhibit the growth of insect pests by preventing metamorphosis [44]. Our results were in favor of caspase-dependent apoptosis with, in particular, the increased caspase-3 activity and the over-expression of p53 and several caspases at the transcriptomic level. The regulation of caspase expression has been shown to be different depending on the chemical compounds that induce apoptosis [41]. In addition, the overexpression of cathepsin B also suggested that lysosomal apoptotic pathways are activated. One gene, DnaJ1, was down-regulated after ACA exposure. DnaJ homolog subfamily A member1 (DnaJ1) belongs to the heat shock 40 kDa protein family, known to play an important role in neuroprotection and hematopoiesis of *Drosophila melanogaster* Meigen [45,46]. Recently, Shu and colleagues [47] discovered a new role for DnaJ1 as an anti-apoptotic protein. In their study, the level of DnaJ1 expression was down-regulated in Sf9 cells undergoing apoptosis following treatment with azadirachtin. When DnaJ1 is knocked out, induced apoptosis is increased. A similar decrease in the mRNA expression of DnaJ1 was observed in our study following exposure to ACA, confirming that ACA induces apoptosis in Sf9 cells.

The present study revealed that ACA from *A. galanga* exhibited an insecticidal activity against *S. frugiperda*. However, to achieve a more efficient integrated pest management, further studies are required to determine the efficacy and stability of ACA in field trails and its effects on natural enemies.

## 5. Conclusions

Fall armywormhas developed resistance to many insecticide molecules. Its invasiveness makes it a major pest worldwide. The invasive populations already present resistance [48], so there is a real need to find new molecules, and ACA could potentially be one of them. It responds to the renewed interest in natural molecules that are not harmful to humans and the environment. This first study on the insecticidal activity of ACA under laboratory conditions is a necessary step for a future optimization and before authorization of its usage in agriculture.

## Figures and Tables

**Table 1:** Primers used in qRT-PCR.

Name	Primer Sequence	Fragment Length (bp)	PCR Efficiencies (%)
Sf-Caspase-1-F	5′-AATGCTGGACGGAAAACAAG-3′	139	99
Sf-Caspase-1-R	5′-AACTGGCATCCTAGCGACAC-3′		
Sf-Caspase-2-F	5′-TCCAGTCCACCCTGATTTTC-3′	83	103
Sf-Caspase-2-R	5′-ACCAAGATCCACGTTTACGG-3′		
Sf-Caspase-5-F	5′-GGCCTCTACGAGTGATGGAC-3′	89	99
Sf-Caspase-5-R	5′-CGGAAGACACGTCAGTCAAA-3′		
Sf-Caspase-6-F	5′-ACCACAAGGAATGGAAGTGG-3′	149	101
Sf-Caspase-6-R	5′-GTGCTGTGTCCGGTACTTCA-3′		
Sf-Cathepsin B-F	5′-AACGGTGACTCCAAAACACC-3′	98	109
Sf-Cathepsin B-R	5′-GAGTACACGTGCTTGCCGTA-3′		
Sf-Cathepsin L-F	5′-AGTGCAGGTACAACCCCAAG-3′	146	100
Sf-Cathepsin L-R	5′-CTGGAAGGTCTCCTGTGAGG-3′		
Sf-DnaJ1-F	5′-TGAGAGAGGGAGGAGTTGGA-3′	93	95
Sf-DnaJ1-R	5′-GTCTACGACCACCGCTGAAT-3′		
Sf-p53-F	5′-GCACTTGATATCGGTGGAGAA-3′	150	96
Sf-p53-R	5′-GATCCTACAGTCACCCAGCA-3′		
Sf-TCTP-F	5′-GGACATCCTTGGCAGGTTTA-3′	147	105
Sf-TCTP-R	5′-TCCTCCTCAAGACCATGCTT-3′		
G6PD-F	5′-GGCCCTGTGGCTAACAGAAT-3′	142	98
G6PD-R	5′-CATCGTCTCTACCAAAAGGCTTC-3′		
L18-F	5′-CGTATCAACCGACCTCCACT-3′	126	108
L18-R	5′-AGGCACCTTGTAGAGCCTCA-3′		
RpL4-F	5′-CAACAAGAGGGGTTCACGAT-3′	149	98
RpL4-R	5′-GCACGATCAGTTCGGGTATC-3′		

G6PD, glucose 6-phosphate dehydrogenase; L18, ribosomal protein; RpL4, ribosomal protein.
